# Supplementary material for: Are carbon emissions trading and green financial instruments synergistic? -Comprehensive quantitative research based on content analysis
Source: PLoS One. 2024 Mar 7;19(3):e0298601. doi: 10.1371/journal.pone.0298601 (PMC10919601; doi:10.1371/journal.pone.0298601)
Supplement: S1 Data — (ZIP) [file pone.0298601.s001.zip › Data/地级市Z_F工作报告.zip!/╜¡╦╒/╗┤░▓╩╨/└┤╫╘╣1⁄2╓┌║┼í╛┬φ┐╦╩2╛▌═°í┐_2016.docx]

各位代表：

现在，我代表市人民政府向大会作工作报告，请予审议，并请各位政协委员和其他列席同志提出意见。

一、“十二五”发展回顾

过去五年，面对错综复杂的宏观环境和艰巨繁重的改革发展稳定任务，我们牢记习近平总书记“把周总理家乡建设好，很有象征意义”的殷切嘱托，在省委、省政府和市委的正确领导下，紧紧围绕建设全面小康社会和苏北重要中心城市两大目标，积极适应经济发展新常态，认真贯彻稳中求进总基调，统筹推进稳增长、调结构、促改革、惠民生、重生态等各项工作，以县为单位达到省定2003版全面小康标准，实现了三次产业比重由“二三一”到“三二一”的历史性转变，实现了人均GDP、城镇化率超全国的重大跨越，胜利完成“十二五”目标任务，为“十三五”乃至更长时期的发展奠定了坚实基础。

五年来，坚持以转型升级为主线，提升了经济发展水平。面对经济新常态，主攻快上重大项目，加速集聚高端要素，顶住经济下行压力，保持了稳中有进、进中趋好的强劲势头。经济总量实现翻番。预计2015年，实现地区生产总值2700亿元，是2010年的1.95倍，人均近9000美元，达到中等收入国家或地区水平;一般公共预算收入350亿元，是2010年的2.5倍;规模以上固定资产投资2203亿元、工业投资1295亿元，分别是2010年的2.4倍和3.2倍;社会消费品零售总额971亿元，是2010年的2倍。优势特色产业发展壮大。“4+2”先进制造业集聚发展，实现产值3500亿元，占比达58%，电子信息产业产值率先超千亿元。“4+3”特色服务业快速发展，物流、电子商务等新兴服务业异军突起，服务业增加值占比年均提高1个百分点，服务业税收占地方税收比重达73.5%。“4+1”现代农业产业加快培育，新增省级以上龙头企业28家，获批6个国家级农业载体，农产品地理标志证明商标数量全国第一，农业综合机械化和粮食收储现代化水平跃居苏北首位，粮食生产实现“十二连增”，农业现代化工程发展指数苏北领先。创新能力显著提升。加快产学研协同创新步伐，建成智慧谷一期工程，签约34家、落户18家著名高校院所研发机构;引进国家“千人计划”专家89名，入选省“双创人才”62名，获批国家级博士后科研工作站5家，建立校企联盟300家，创成国家级大学科技园区。国家级高新技术企业增加到246家，是2010年的5.8倍，高新技术产业产值占比达26%，全社会研发投入占GDP比重达1.7%，科技进步贡献率达51%，科技创新成为产业升级的重要支撑。

五年来，坚持以中心城市为引领，加快了新型城镇化步伐。积极构建“一主四副多点网络化”的新型城镇体系，不断优化城乡空间形态，实现了工业化和城镇化互动并进、产业与人口同步集聚。中心城市品质显著提升。突出规划引领，滚动实施“十项重点工程”，加快推进“一廊四区多片”建设，累计投入3891亿元、实施了4150个城建重点项目，中心城市框架业已形成，“六大功能”全面提升，建成区面积达155平方公里、人口增至140万人。开工建设连淮扬镇、徐宿淮盐两条铁路，奠定了区域综合交通枢纽地位。建成万达广场、体育中心、“四馆”、大剧院等一批重大功能性项目和地标性建筑，完成南水北调东线一期、盐河航道“五改三”、入江水道整治和淮金公路等重大基础设施工程，实现了县县通高速。深入开展城市环境综合整治，连续实施道路通达、绿色小客厅、老旧小区改造、农贸市场提升等项目，积极推进智慧城市建设，顺利通过国家卫生城市复审，文明城市创建取得重要进展，城市品位进一步提升。城乡统筹步伐加快。四座县城功能不断增强，10个省级重点中心镇建设取得突破，一批特色镇加快培育，常住人口城镇化率达58%，五年提高7.4个百分点，实现了常住人口止跌回升的重大转折。优化乡村布局规划，加快重点村和特色村建设，在苏北率先以市为单位完成村庄环境整治;实施新一轮区域供水工程，解决了119万农村居民饮用水安全问题;推进农村公路建设，实现行政村客运班车、乡镇垃圾转运和污水处理设施全覆盖。生态文明特色彰显。在全省率先编制实施主体功能区规划，加强生态环境保护和污染防治，城市绿化覆盖率达41%，获批全国生态文明先行示范区和水生态文明试点市，创成苏北首个省级生态市、即将迎来国家技术评估。积极推进绿色低碳循环发展，获批国家低碳试点城市、新能源示范市、可再生能源建筑应用示范市。单位GDP二氧化碳排放五年下降23%，市区PM2.5浓度均值与2013年相比下降26.6%，绿色生态已经成为淮安最靓丽的城市名片。

五年来，坚持以台资高地为特色，拓展了开放合作空间。不断扩大视野、拓宽领域、提升层次，开放工作实现了逆势攀升、跨越突破。台资高地加速崛起。创新实施“台资项目倍增、交流平台扩展、101%服务升级”三大计划，获批全国台资企业产业转移集聚服务示范区，落户大陆首个两岸信息家电产业园，连续四年获评台商投资“极力推荐城市”。新批台资项目428个，台企总数近1200户，实际利用台资33.2亿美元，一批台资龙头项目成为产业集聚发展的重要支撑。对内对外开放质态不断提升。淮安出口加工区升级为国家级综保区，淮安机场一类航空口岸获批开放，增创了开放合作新优势。强化产业链招商、专业招商和驻点招商，累计引进投资超3000万美元外资项目141个，其中投资超1亿美元项目36个，实际利用外资57.5亿美元，总量居苏北第二，创成省级利用外资转型发展示范区;累计引进投资超亿元内资项目1050个，其中投资超10亿元项目142个。对外贸易年均增长13.6%，高于全省10.5个百分点。新缔结国际友城和友好交流城市25对，对外合作交流遍及五大洲。区域合作全面深化，省级以上开发区实现南北共建园区全覆盖，实施了宁淮现代服务业集聚区、新兴科技产业园等合作项目。园区承载能力明显提高。淮安经济技术开发区综合实力进入全省20强，建成12个省级特色产业园区，规划建设了8个台资产业园和7个重点国别园区。全市省级以上开发园区集聚规模以上工业企业1600多户，成为产业发展的主阵地。

五年来，坚持以重点领域改革为先导，增强了经济社会发展活力。共有10项改革列入国家试点，14项改革列入省级试点，形成了富有淮安特色的改革体系。行政审批制度改革走在前列。在全国率先开展“先照后证”工商登记制度改革，在全省首家公布政府部门权责等5张清单，建立“1+7”事中事后监管体系，盱眙相对集中行政许可权改革试点成为全国样本，政务服务工作全省领先、全国一流。改革有效激发了“全民创业、淮商崛起”的活力，全市私营企业、个体工商户分别达6.4万户和22万户，是2010年的2倍和1.4倍。土地使用制度改革取得突破。改革方案得到了国土资源部、省政府和省国土资源厅的大力支持，编制实施了首批5个功能片区土地利用规划，洪泽成为全省首家建设用地“双减量”试点县。去年用地指标比上年增加了12.5%，土地流转、规模经营面积比重分别比上年提高了10个和9个百分点。金融改革不断深化。今世缘酒业、井神盐化成功在A股主板上市，累计有32户企业登陆多层次资本市场，共引进金融机构39 个，农村金融综合服务站实现行政村全覆盖，探索开展农村承包土地经营权抵押贷款试点。2015年末，金融机构人民币存贷款余额分别达2329亿元和 1865亿元，是2010年末的2倍和2.2倍。新型城镇化综合配套改革扎实推进。积极开展“多规合一”试点，实施金湖全域城镇化、淮阴区产镇融合和马坝镇强镇扩权改革，出台实施户籍制度改革意见，积极稳妥推进洪泽等地乡镇行政区划调整，促进了产业和人口向城镇集聚。进一步深化医药卫生体制改革，城市公立医院全部实行药品零差率销售，全面推开分级诊疗试点。统筹推进教育、文化、科技、国企、农业农村等领域改革。

五年来，坚持以改善民生为根本，提高了人民群众的幸福指数。坚持把新增财力的四分之三以上用于改善民生，持续实施“十件实事”等民生幸福工程，城乡居民收入分别达28120元和13210元，是2010年的1.6倍和1.8倍。社会事业协调发展。学前三年教育毛入园率提高到98.8%，在苏北率先通过义务教育发展基本均衡国家督导认定，高考二本以上录取率连续六年保持苏北第一，全面实现教育基本现代化，构建了现代职业教育体系。建成国家级群众满意乡镇卫生院11个，三甲医院达到5所，人均期望寿命由76.75岁增长到78.16岁，成为全国创建幸福家庭活动试点市。大运河成功入选《世界文化遗产名录》，获得全国优秀儿童文学奖、全国戏剧梅花奖等多项大奖，实现乡镇文化站、村居文化室、农家书屋全覆盖;成功承办第十八届省运会，建成省公共体育服务体系示范区。社会保障水平稳步提高。基本养老保险、医疗保险和失业保险参保覆盖面均达95%以上，城乡低保标准分别从300元、160元提高到490元、370元，城镇居民医保年度最高支付限额调整到25万元。多渠道解决群众住房困难，共有产权住房制度列入全国试点，新建保障性住房803万平方米，累计发放住房公积金贷款89.6亿元。在全国首创建设“关爱驿站”，城乡社区居家养老服务中心覆盖率分别达到100% 和80%。创建“阳光慈善”品牌，建立一门受理、协同办理的社会救助工作机制。就业创业实现城乡统筹，新增就业33.9万人，扶持创业7.9万人，成为省级创业型城市。全面完成人均年收入低于4000元人口脱贫任务。社会治理创新不断加强。建立健全“一委一居一站一办”新型社区服务管理体制，城乡社区网格化治理体系更加完善，县乡村联通共享的综治信息化系统全面建成，社会管理创新工程发展指数全省第一。全面启动“六位一体”社会治理体系建设，推行城区“网格化”、农村“一公里警务圈”社区警务模式，“阳光警务”在全省首家推出网上交通安全综合服务，群众安全感、满意率保持在95%以上。“阳光信访”、 “1+3”安全生产监控模式、透明食药监等经验在全国推广，“一案三制”应急管理体系进一步完善，社会总体保持和谐稳定。其他各项工作协调推进。军民融合发展不断深化，圆满完成征兵任务，有望实现全国双拥模范城市“四连冠”。援疆工作走在全疆、全省前列，累计投入5.6亿元实施援建项目50个。统计、物价、供销、质监、海关、检验检疫、机关事务等工作取得了新成绩，广播电视、民防、气象、地震、邮政、仲裁、档案、地方志、民族宗教、老龄、妇女儿童、青少年、残疾人、关心下一代、红十字等事业实现了新发展。

五年来，坚持以人民满意为追求，提高了政府执行力和公信力。与时俱进加强政府自身建设，以担当的精神、过硬的本领、务实的作风更好地引领和推动发展。注重统筹谋划。加强对全局性、长远性问题的研究谋划，努力提高工作的系统性、前瞻性和指导性。充分发挥规划的引领作用，先后编制了主体功能区、盐化新材料产业园、里运河文化长廊等44项综合性和专项规划，切实以科学规划提升决策水平、增强投资信心、引领跨越发展。突出创新作为。在优势特色产业培育、新型城镇化、重点领域改革和生态文明建设等方面建立了新体系，在优化投资环境、推动全民创业、促进土地集约节约利用等方面出台了新政策，在推进县域经济跨越崛起、提高招商效率、集聚创新资源、促进园区提档升级等方面推出了新举措，增创了优势、破解了难题、激发了活力。坚持依法行政。构建了法治政府建设制度体系，完善重大行政决策程序，健全政府法律顾问制度，加强行政执法规范化建设，强化行政复议应诉工作，深化政务公开，全面推行行政权力网上公开透明运行，顺利通过“六五”普法考核验收。自觉接受市人大的法律监督、工作监督和市政协的民主监督，开通“代表委员直通车”，人大代表建议和政协提案办理满意率均达 99%以上。持续改进作风。认真落实中央“八项规定”精神，深入开展党的群众路线教育实践活动和“三严三实”专题教育，完善公开承诺、首问负责和挂钩帮扶等机制，扎实推进“两为”专项整治，加大行政问责力度，提高了行政服务效能。严格落实党风廉政建设“两个责任”和“一岗双责”，全面推进惩治和预防腐败体系建设，切实加强行政监察和审计监督，坚决惩治腐败问题，努力形成风清气正的政风新常态。

各位代表，刚刚过去的2015年，我们牢牢把握“上水平”工作主线，迎难而上，奋力拼搏，克服了超出预期的挑战，全面完成市七届人大四次会议确定的各项任务，为收官“十二五”画上了圆满的句号。经济发展稳中趋好。预计GDP、规模以上固定资产投资、一般公共预算收入、城镇和农村居民人均可支配收入同比分别增长10%、22.7%、13.5%、9%和10%，投资、消费、收入等支撑性指标增幅在全省位次不断前移，高新技术产业产值增速快于工业增速4个百分点，技改投入占工业投资比重提高了10个百分点，财政收入结构持续优化。项目建设势头强劲。呈现了数量多于上年、体量大于上年、开工率高于上年、进度好于预期的特点，淮安工业史上单体投资最大的富强新材料以及中兴智慧产业园、臻鼎科技等一批重大项目开工建设，纳沛斯等一批重大项目竣工投产，现代有轨电车一期工程建成运营，古淮河西游记文旅区、里运河文化长廊部分景区建成开放，白马湖国家湿地公园获批建设，基本完成西南化工区居民搬迁。重点工作亮点纷呈。淮河入海水道二期同步实施二级航道工程获国家主管部门同意，淮台直航正式开通，二类水路口岸通过省验收，立体式对内对外开放通道正在加快形成。周恩来故里旅游景区创成苏北首批国家5A级景区，获批国家电子商务示范基地、省新能源汽车产业基地，发展平台的集聚力不断增强。着眼“十三五”规划发展，精心编排了 60多个重大事项，加大向上争取力度，其中淮河生态经济带上升为国家战略、融入江苏沿海开发战略、建设宁淮铁路等重大事项，得到了上级支持、列入了相关规划，将极大拓展淮安未来的发展空间。

回顾“十二五”的发展历程，成绩来之不易，经验弥足珍贵。在科学跨越发展实践中，我们始终保持定力勇担当。面对淮安发展不足的问题，紧抓发展第一要务不动摇，咬定各项目标不放松，坚持谋在高处、干在实处、抓在关键处，保持了主要经济指标增幅连续10年位居全省前列，开创了科学发展的新局面。我们坚持深化改革增优势。面对复杂严峻的发展环境，奏响改革最强音，一系列改革列入国家和省级试点，创造了许多生动鲜活的淮安经验，拓展了后发地区跨越赶超的新空间。我们突出主攻项目强支撑。面对产业层次不高、制约瓶颈较多的实际，牢固树立“项目为王”理念，以锲而不舍的盯劲向上争取项目，以百折不挠的韧劲对外招引项目，一个又一个重大突破让梦想变成现实，一个又一个重大项目落地由不可能变成可能，增强了稳增长、调结构的新动能。我们牢记发展为民增福祉。面对群众不断增长的新期盼、新诉求，倾尽全力做好富民、惠民、安民、乐民之事，努力以干部的辛苦指数换取群众的幸福指数，提高了人民群众的获得感。

各位代表，“十二五”淮安发展取得了令人鼓舞的辉煌成就，这是省委、省政府和市委正确领导的结果，是市人大、市政协监督支持的结果，是全市人民团结奋斗的结果，也得益于历届领导班子打下的良好基础。在此，我谨代表市人民政府，向广大干部群众，向人大代表、政协委员和离退休老同志，向市各民主党派、工商联、无党派人士和各人民团体，向驻淮部队、武警官兵和部省驻淮单位，向来淮投资创业者，向所有关心和支持淮安发展的海内外朋友，表示衷心的感谢并致以崇高的敬意!

在肯定成绩的同时，我们也清醒地认识到，在加快经济发展中还面临一些严峻的挑战，在加强自身建设中还存在一些突出的问题。主要表现在：经济下行压力持续加大，企业运行困难增多，有效投入还不足，稳增长、保前列的任务十分艰巨;制约发展瓶颈还比较多，新兴产业竞争力不强，促改革、调结构仍需大力攻坚;城乡居民增收难度加大，基本公共服务均等化水平有待提升，社会矛盾和安全隐患仍然较多，惠民生、促和谐与群众的期盼还有差距。面对这些挑战，需要我们积极应对、创新作为，不断加以克服。引领新常态的能力还不强，不敢为、不善为甚至乱作为现象仍然存在，服务发展的效能还不高，依法行政能力有待进一步增强。针对这些问题，我们将高度重视，采取有效措施，切实予以解决。二、“十三五”主要任务

各位代表，“十三五”是淮安全面建成小康社会的决胜阶段，也是奠定苏北重要中心城市地位的决战阶段，必将是令人期待、催人奋进、大有可为的又一发展黄金期。面对新形势、新任务，我们要在适应新常态中把握大趋势，在应对新挑战中争取主动权，在发挥新优势中谋求新跨越，不断拓展新空间、开创新局面。根据《中共淮安市委关于制定淮安市国民经济和社会发展第十三个五年规划的建议》，市政府制定了“十三五”总体规划纲要(草案)，提请大会审议。纲要(草案)全面提出了我市“十三五”时期经济社会发展的指导思想、总体目标、主要任务和重大举措，经本次大会审议批准后，市政府将作为施政依据，认真付诸实施。

“十三五”时期，我们将高举中国特色社会主义伟大旗帜，深入贯彻落实党的十八大、十八届三中四中五中全会和习近平总书记系列重要讲话精神，秉持创新、协调、绿色、开放、共享发展理念，以“牢记总书记嘱托，建好周总理家乡”为引领，以“打造增长极，共筑崛起梦”为统揽，以提高发展质量和效益为中心，以保障和改善民生为根本，大力实施创新驱动、工业强市、开放引领、全民创业、城乡一体化、绿色发展战略，向着更高层次科学跨越发展奋力迈进，谱写好“中国梦” 的淮安篇章。

“十三五”时期的奋斗目标是：全面建成小康社会，奠定苏北重要中心城市地位，打造江苏发展新兴增长极。经济实力强，到2020年，地区生产总值年均增长10%左右，力争人均达到9万元，初步达到中等发达国家或地区水平;一般公共预算收入年均增长10%;工业增加值和社会消费品零售总额年均增长11%左右，三次产业比重进一步调整为9:43:48，全社会研发投入占GDP比重达2.3%，科技进步贡献率达 60%，经济发展质量显著提升。百姓生活富，城乡居民收入年均分别增长8%和8.5%左右，城镇登记失业率控制在4%以内，城乡基本社会保险覆盖率达 97%，常住人口城镇化率达63%，基本公共服务水平全面提升，群众获得感和幸福感明显增强。生态环境美，建成全国生态文明先行示范区，单位GDP能耗、水耗下降和二氧化碳、主要污染物减排完成省定指标，林木覆盖率稳定在26%，村庄环境整治达标率为100%，城乡形态不断优化，人居环境显著改善。社会文明程度高，法治政府基本建成，民主法制更加健全，社会治理全面加强，社会主义核心价值观深入人心，公民思想道德和科学文化素质明显提高，社会文明程度测评指数达90%，努力创成全国文明城市，社会更加和谐稳定。

实现“十三五”的发展蓝图，将在八个方面求突破：建设淮安都市区。从战略和全局的高度，优化中心城市空间布局，实现“纳湖入城”;坚持片区联动、组团相间、产城融合，推进“一核五区”建设，不断增强集聚功能;加快东融西拓、南联北接步伐，成为国家战略的一个重要节点城市;突出中心城市引领带动，加快形成城乡一体化发展新格局。完善现代产业体系。打造“4+2”先进制造业升级版，四大主导产业产值均过千亿，两大战略性新兴产业产值实现翻番，成为长三角北部先进制造业基地;推进“4+3”特色服务业高端化发展，加快建设长三角知名旅游目的地城市、区域性物流中心城市、国家电子商务示范城市和长三角北部滨湖休闲养生基地，构筑苏北现代服务业高地，为经济转型升级提供动能;推进“4+1”现代农业提质增效，建设国家现代农业示范区，基本实现农业现代化。建成综合交通枢纽。以两条高速铁路和淮河入海水道二级航道建设为契机，构建以高速铁路为骨干、国际空港为优势、高等级公路为支撑、内河航运为特色的现代综合交通体系，加快建设空港产业园、高铁新区、港口物流园，大力发展枢纽经济。提升对外开放水平。全面推进国家级台资企业产业转移集聚服务示范区建设，积极拓展欧美日韩等外资招商领域，深化宁淮、淮河沿线等区域合作，优化面向国际国内的开放环境和平台，形成全方位、多层次、宽领域的开放新格局。成为重点领域改革示范区。以列入国家和省试点的重点领域改革为引领，勇于先行先试，营造浓厚的改革创新氛围，推动更多的改革开花结果，创造出更多的淮安特色经验，释放出更大的改革红利。打造“双创”高地。实施“互联网+”行动计划，推进大众创业、万众创新，加快品牌建设，加速淮商崛起，全面提升民营经济发展水平，积极构建具有淮安特色的区域创新体系，创建国家创新型城市，使淮安成为区域性创新创业高地。做优生态文化品牌。深化生态文明体制改革，加快建设全国生态文明先行示范区、国家低碳城市和国家水生态文明城市，大力发展绿色循环经济，努力使绿水青山变成金山银山;发挥文化的引领力和感召力，加强文化遗存保护，健全现代公共文化服务体系，推进文化产业发展，努力让良好生态和特色文化成为淮安最响亮的品牌。增强人民群众获得感。紧紧围绕“七个更”目标，千方百计促进就业，形成更加公平可持续的社会保障制度，健全基本公共服务体系，打好新一轮脱贫攻坚战，使广大人民群众共享全面小康社会建设成果。三、2016年重点工作

今年是实施“十三五”规划的开局之年。我们将认真贯彻党的十八届五中全会、省委十二届十一次全会和中央、省经济工作会议精神，按照市委的总体部署，把握稳中求进总基调，适应经济新常态，注重供给侧结构性改革，突出去产能、去库存、去杠杆、降成本、补短板五大任务，心无旁骛谋发展，全力以赴抓落实，为实现“十三五”发展目标开好局、起好步。

今年全市经济社会发展主要预期目标是：地区生产总值增长10%左右，规模以上工业增加值增长11%，一般公共预算收入增长11%左右，规模以上固定资产投资和工业投资分别增长18%左右和20%，社会消费品零售总额增长11%左右，注册外资实际到账增长5%，城乡居民人均可支配收入分别增长8.5%和 9.5%，城镇登记失业率控制在4%以内，居民消费价格指数不超过全省平均水平，节能减排完成省定任务。

围绕上述目标任务，将突出抓好以下六个方面：

(一)千方百计稳增长，保持经济健康发展。坚持项目为王、企业为主，着力优化投资、扩大消费和提升外贸，协同拉动经济平稳健康发展。

发挥投资关键作用。突出项目主抓手，扩大有效投资，完成规模以上固定资产投资2580亿元，为稳增长提供持续动力。落实“一考二报三单五制”推进机制，加快实施220个重大项目，全年完成投资1072亿元。强化财政资金撬动功能和金融信贷保障作用，整合充实产业引导资金，大力推广PPP模式，带动更多社会资本参与投资。

发挥消费基础作用。强化政策引导，培育新的消费热点，推动消费升级，扶持发展旅游、健康养生等新兴业态，鼓励发展分享经济，消费对经济增长的贡献率提高到52%。挖掘传统消费潜力，引导传统商贸企业发展连锁经营，积极拓展农村消费市场，培育一批餐饮精品名店，做实淮扬菜品牌。实施住房保障货币化补贴、棚户区改造货币化安置，促进房地产去库存。引导市场转型升级，探索设立大宗商品交易市场，争取列入全省进口商品交易中心试点。规划建设特色商业街区，满足群众多层次的消费需求。

发挥外贸拉动作用。引导企业开辟国际市场，实现进出口总额增长5%。实施外贸“双百工程”，大力扶持成长型外贸企业，新培育进出口企业100户、进出口超亿美元企业2户。深入挖掘农产品、食品等特色产业出口潜力，农产品出口增长18%以上。优化外贸结构，推动加工贸易转型升级，积极发展一般贸易和服务贸易，拓展保税物流、保税加工业务，提高外贸进出口贡献份额。大力发展跨境电商业务，充分发挥外贸综合服务平台和进口代理平台作用，拓宽外贸营销渠道。

发挥企业支撑作用。按照提升重点企业、扶持成长型企业、鼓励发展小微企业的思路，强化挂钩帮扶、政策支持和要素保障，帮助企业解决实际困难。积极提升重点企业运行质态，对100户骨干企业落实一企一策帮扶机制，实行一户企业一个工作组、一张服务清单，支持企业实施技术改造和战略重组，力争销售超10亿元企业达50户。发挥“六大服务平台”作用，促进中小企业降成本、增效益，新增销售超亿元企业30户。大力推进大众创业、万众创新，全年新增私营企业 1.15万户、个体工商户2.7万户，让淮安成为创客群涌、企业群聚的新高地。

(二)坚定不移调结构，加快产业转型升级。突出科技创新驱动，健全现代产业体系，强化主导产业支撑，促进经济保持中高速增长、产业迈向中高端水平。

强化科技创新驱动。坚持把科技创新作为产业升级的主引擎，设立产业创新发展基金，突出企业主体地位，加快产业技术协同创新联盟建设，重点培育创新型领军企业10户、省级以上高新技术企业280户，高新技术产业产值占比达27.5%，万人发明专利拥有量超过3件，进入国家创新型试点城市行列。做强园区创新平台，推动淮安高新区申创国家级高新区、盐化新区申创省级高新区。强化人才支撑，新引进国家“千人计划”人才项目20个、省“双创”人才项目15个。

加快发展先进制造业。顺应互联网等新技术和产业变革新趋势，引导制造业朝着分工细化、协作紧密方向发展。新开工投资超亿元项目150个，新竣工设备投资超千万元项目100个，培育省市“两化融合”示范试点企业60户。重点推动“4+2”产业建链强链补链，力争全年产值突破4000亿元。加快建设富强新材料、华昌新材料、澳洋顺昌集成电路和清浦生物医药产业园等一批重大项目，力争竣工实联化工医药用盐、臻鼎科技等项目，加速产业集群集聚发展。发挥资源和品牌优势，提升今世缘酒业、旺旺食品、双汇食品等龙头企业整体竞争力，食品产业产值超千亿元。全力扶持敏安电动汽车整车生产项目实施，打造全国重要的新能源汽车研发、生产基地。

培育壮大特色服务业。围绕打造“六在淮安”品牌，完善“4+3”特色服务业产业发展规划，制定实施意见，进一步整合优化全产业链，加快宁淮现代服务业集聚区等重点园区建设，提升服务业发展水平。依托综合交通优势，加快建设传化物流港等项目，大力发展商贸、快递、港口、保税和仓储等物流业态。放大生态文化优势和淮扬美食特色，统筹推进里运河、古淮河和白马湖、洪泽湖“两河两湖”板块建设，加快发展旅游、健康养生和文化创意产业，打响淮安文旅品牌。加快淮安电子商务现代物流园等平台建设，促进传统产业与电子商务深度融合，创建国家电子商务示范城市。

做优做特现代农业。聚焦“4+1”现代农业产业提质增效，推进“接二连三”发展。提升项目带动力，引进实施投资超亿元农业产业化项目10个、超 3000万元农产品加工项目35个，新增家庭农场400个，每个县区建设2-3个升级版乡村旅游点和农产品电商示范点。提升园区承载力，重点加快3个国家级、10个省级农业园区建设，推进淮金路、黄河故道沿线、环白马湖等重点区域串点成线和扩面成带。提升基础设施保障力，完成分淮入沂整治、黄河故道综合开发等工程，不断完善农田水利基础设施，集中连片推进高标准农田建设。提升科技服务支撑力，建成高分子水稻选育基地等一批重点项目，加快“淮安大米”产业发展，推动农机装备结构优化升级，大力发展就近就地粮食烘干中心，提高气象为农服务水平。

(三)深化改革促开放，增强发展内在活力。推进重点领域改革落地生根，扩大对内对外开放领域，最大限度地释放改革红利，增创开放新优势。

推进新型城镇化综合试点。探索完善新型城镇化和城乡发展一体化的有效路径，突出试点重点，努力打造全国新型城镇化建设示范区。深化“多规合一”改革，加快空间信息管理平台建设，建立全市统一的空间规划体系，提升产城融合水平。实施新一轮行政区划调整，积极推进洪泽撤县设区、清河和清浦合并设立清江浦区，稳妥开展乡镇布局调整，不断优化市域城镇空间形态。扎实开展涟水、洪泽、金湖、盱眙马坝等国家新型城镇化综合试点工作，有序推进农业转移人口市民化，逐步将进城落户务工人员纳入城镇社保体系和住房公积金覆盖范围，切实让他们能够落下户、生活好。

放大特色改革试点效应。按照总结经验、扩大试点、健全机制的总体要求，推动改革全面开花结果。纵深推进行政审批制度改革，总结推广盱眙经验，组建市县两级行政审批局，全面实施行政许可权相对集中，优化再造建设项目审批流程，探索试点“互联网+政务服务”;加大综合行政执法改革力度，完善市场综合监管体系。扩大土地使用制度改革试点范围，扎实推进五个功能片区试点，有序开展其他功能片区土地利用规划编制和实施工作;加快农村土地产权确权发证，基本完成承包经营权确权登记颁证，新增土地股份合作社100个以上。深化金融改革，支持企业对接资本市场，力争8户企业挂牌上市;推动银行间市场直接债务融资，力争融资100亿元以上，有针对性地发展融资担保业，完成金融控股集团组建工作;加大金融机构引进力度，推动南京银行淮安分行尽快开业，有序发展小贷公司。协调推进城市公立医院、供销社、国有粮食企业、物价体制等改革，完成公共交通和小型水利工程管理体制改革。

扩大对内对外开放。围绕打造台资高地升级版，继续实施台资项目倍增等“三大计划”，编制实施台资企业产业转移集聚服务示范区总体规划和行动方案，积极争取省出台专项支持政策。用好用足中韩、中澳等自贸区政策，拓展招商领域，创新招商方式，主攻产业链项目招引，强化外资招商组织和考核，各县区分别引进欧美日韩项目3个、新批1个以上超亿美元外资项目，淮安经济技术开发区分别引进5个以上，力争在10亿美元以上重大项目招引上取得新突破;新引进投资超亿元内资项目180个以上，其中超10亿元重大项目10个。完善开发园区产业配套功能，引进和打造一批设计、研发、检测等公共服务平台。支持涟水、盱眙和淮安区创建国家级经济技术开发区，推动清河新区、施河现代教育装备产业园等申创省级开发区，重点打造1-2个中外合作园区。鼓励企业开展境外投资，加快淮安企业国际化步伐。推进国际友城拓展工程，不断优化对外开放环境。

大力发展枢纽经济。充分利用逐步凸显的区域性综合交通枢纽优势，加快各类要素资本集聚。启动建设高铁新区，统筹推进站场基础设施建设、产业规划编制和重大项目招引，加快打造高铁商务商业圈。依托空港产业园建设，发展航空航运枢纽经济，推动航空运输、航空制造等重点产业集聚，加快淮安电子口岸建设，开通国际航空货运业务。围绕内河航运和高等级公路网络体系，推进沿河港口产业园和沿路现代物流产业园建设，大力发展集散分拨、仓储配送等高附加值产业。

(四)一着不让抓统筹，促进城乡共建共享。强化规划引领，加快重点工程项目建设，推动中心城市强功能、县域发展上水平、重点城镇见形象。

增强中心城市功能。围绕提升“四个力”，完成新一轮城市总体规划修编，投资660亿元，实施780个城建重点项目。重抓“十项重点工程”建设，里运河文化长廊开工建设中国漕运城、老坝口历史风貌区等一批重点项目，古淮河西游记文旅区建成龙宫大白鲸嬉水世界主体工程，淮安综合大市场开工建设现代物流园项目，白马湖森林公园基本建成菊花园、红叶谷等核心区工程，金融中心一期工程部分主体竣工，完善智慧谷一期功能配套，推动已入驻机构发挥功效。继续推进重点功能片区规划建设，提升里运河文化长廊的整体影响力，提高淮海中心商业区、水渡口中央商务区、河下文旅开发区、生态新城绿色低碳示范区的容纳能力，加快 “纳湖入城”步伐。更加注重城市形象打造，推进精细化管理，启动省级人居环境奖创建，加强控源截污、地下管廊、海绵城市等建设，继续开展棚户区、老旧小区改造和后街背巷整治，推进智慧城市建设和全国文明城市创建。

加快重大基础设施建设。全面推进连淮扬镇、徐宿淮盐高铁建设，启动建设高铁客运枢纽;推进淮安机场二期扩建工程，积极开辟国际航线;加快348省道等工程建设，完善国省干线公路网络;实施淮河出海航道整治和新港二期等项目，加强与沿江沿海港口的互联互动，提升二类水路口岸的辐射力。实施城市道路通达工程，渠化改造韩侯大道、黄河东西路;规划建设城市快速交通系统，努力打造一刻钟城市通勤圈。实施河湖连通工程，健全水安全保障体系，不断强化水利对区域经济社会发展的支撑作用。

推动县域经济加速发展。抓好片区规划建设，推动基础设施共建共享，促进人口和产业集聚。重抓10个重点中心镇建设，基本建成“十个一”工程项目，按照 “一镇一品”要求扶持建设一批特色名镇，加强连接县域重要经济节点、港口、旅游景区道路建设。突出产业支撑，坚持精准定位、错位发展，充分挖掘淮安区古城历史文化底蕴和区位优势，发展物流、电子商务和文化创意等业态，促进淮阴区“一区五园”产城融合互动发展，积极支持涟水融“市”入“海”、盱眙撤县建市，进一步彰显洪泽魅力湖滨新城、金湖水乡园林城市韵味，加快发展健康养生等新兴服务业。深化“一县一策、一县一题”专项扶持，进一步完善帮扶机制，加大工作力度，推动县域经济加速突破。发挥特色工业园区发展基金引导作用，力争首批10个市级特色工业园开票销售总额超150亿元。

(五)持之以恒重生态，扎实推进绿色发展。把生态文明建设融入经济社会发展全过程，坚持源头严防、过程严管、后果严惩，坚决呵护好淮安人民的美好生活家园。

加强生态文明建设。以建设全国生态文明先行示范区为主线，强化生态红线刚性约束，推进生态约束与多规融合相衔接，严格环评审批和“三同时”制度，不断提高生态文明建设水平。加大重点领域环保力度，加强城乡治污设施和生活垃圾收运处置体系建设，规范农贸市场管理，严格固体废弃物安全处置;加快建设低碳交通、低碳园区和低碳社区，提升百户重点用能企业能效。

重抓水气污染防治。贯彻落实国家“水十条”，合理划分地表水生态环境功能区，实行排污许可管理，健全环境影响评价等制度，重点推进洪泽湖、白马湖水质良好湖泊保护，确保白马湖保护项目通过国家环保部考核验收。加强大气污染防治，实施火电行业超低排放、非电力行业脱硫脱硝和除尘改造工程，综合治理燃煤小锅炉、挥发性有机物、扬尘污染和机动车船尾气，推广应用新能源汽车，确保PM2.5浓度下降、良好天气增加。

着力打造宜居家园。完成全市镇村布局规划，实施重点村和特色村建设示范工程，促进“美丽乡村”建设提标升级，打造市级康居乡村30个。继续实施农村公路提档升级工程，新改建道路300公里、桥梁45座。按照新型社区标准，提升基础设施配套和公共服务保障水平。健全村庄环境整治长效管护机制，全面推进秸秆禁烧和综合利用，让群众享有天蓝水净、地绿山青的生态自然环境。

(六)加大投入惠民生，不断增进群众福祉。突出全覆盖、保基本、多层次、可持续，加大社会事业和民生投入，完成投资55.6亿元，兴办民生十件实事，促进社会和谐稳定。

协调发展社会事业。加大教育现代化建设力度，促进各级各类教育优质均衡发展，新扩建一批省优质幼儿园，推动淮阴商校等中职校升格，加快建设恩来干部学院核心区，完成淮安开放大学主体工程。推进“健康淮安”建设，深化医药卫生体制改革，积极构建现代医疗卫生体系，实施5000名基层医学人才培养工程，完善分级诊疗制度，积极扩展妇产儿童医疗资源，落实全面两孩政策，促进人口均衡发展。加快建设国家级公共文化数字服务平台、省级公共文化服务体系示范区、县区文化“三馆”，举办第二届百姓文化艺术节，力争推出一批具有全国影响的文艺名家名作。广泛开展全民健身运动，促进教体融合，办好市第八届运动会。做好国防教育和双拥工作，加强基层人防机构和队伍建设，推进军民融合深度发展。

完善社会保障体系。健全促进就业创业体制机制，继续将高校毕业生就业放在首位，坚持兜底保障就业，实现新增就业6万人。大力实施全民参保登记计划，加快建立城乡统一的居民基本养老和医疗保险制度，稳妥推进机关事业单位养老保险制度改革，完善被征地农民刚性进入社会保险机制和城乡居民基本养老保险参保缴费激励机制。健全大病保险制度，减轻大病患者医疗费用负担。持续实施精准扶贫，统筹安排帮扶项目和资金，促进帮扶对象形成可持续脱贫能力。建立“救急难” 预警机制，建成“大救助平台”，落实残疾人补贴政策，普及推广农村“关爱驿站”，加快建设多层次养老服务体系。打造透明放心菜市场、透明安全餐饮，创建食品安全城市。

维护社会和谐稳定。切实加强新一轮平安淮安、法治淮安建设，推动“六大中心”规范化运行，拓展“阳光警务”内涵，构建立体化现代化社会治安防控体系。推进社会治理精细化发展，完善三级综合管理服务平台，深化城乡社会治理“两网融合”，促进稳评工作向基层延伸，全面开展社会治安综合保险工作，健全社会矛盾排查预警和调处化解综合机制。启动实施“七五”普法教育，增强群众法律意识，保护群众合法权益。推进城乡和谐社区建设，鼓励支持社会组织参与社会治理，完成第六届社区委员会、第十一届村委会换届选举工作。严格落实安全生产责任制，探索建立公共安全源头管理机制，进一步健全事故灾难、暴力恐怖活动预警预防和应急处置体系，提升防范管控各类风险能力。

各位代表，“天下难事必作于易，天下大事必作于细。”开启“十三五”新征程，使命光荣，任务艰巨。我们将以实现好、维护好、发展好人民群众的根本利益为出发点，以提高新常态下的履职能力为着力点，切实加强自身建设，努力打造服务型、效能型、法治型政府。坚持科学施政，营造求新、求快、求优的工作导向。围绕既定目标，突出重要领域、重大项目和重点工程，以新思路、新举措、新机制，打好改革创新“组合拳”，破解发展中的困难挑战和瓶颈制约。大力弘扬“马上就办”的作风，做到雷厉风行、立说立行，在紧张快干中抢占先机、赢得主动。以敢为天下先的勇气和永不满足的精神，推动各项工作快中求好、好中求优，确保在新一轮的区域发展中进位争先。坚持务实理政，健全会办、帮办、督办的推进机制。强化实干为先理念，进一步健全定期会办和联席会议等制度，加大重点工作统筹协调力度。进一步完善领导干部挂钩帮扶、责任包干制度，形成以上率下、齐抓共管的工作机制和责任体系。进一步强化效能监察，加大行政问责力度，营造争先恐后抓落实的浓厚氛围，不断提高政府的执行力和公信力。坚持依法行政，打造公开、公平、公正的政务环境。完善依法行政制度体系，加强政府规章和规范性文件制定工作，提高依法办事能力，严格规范公正文明执法，让群众感受到法治的力量、正义的温暖。落实各类权责清单，健全决策、执行、监督机制，自觉接受人大法律监督、政协民主监督和社会舆论监督，保证权力运行规则公开、程序公开、过程公开、结果公开。坚持从严治政，树立清正、清廉、清明的政府形象。严守政治纪律和政治规矩，认真落实党风廉政建设责任制，严格履行“一岗双责”，全面贯彻《廉洁自律准则》和《纪律处分条例》。秉持勤勉奉献、厉行节约，严控“三公”经费，守住廉洁底线，以零容忍无禁区全覆盖的态度严惩腐败，切实遏制不正之风和腐败问题，努力实现干部清正、政府清廉、政治清明。

各位代表，宏伟的目标激励我们团结奋斗，美好的蓝图需要我们同心绘就。让我们紧密团结在以习近平同志为总书记的党中央周围，在省委、省政府和市委的正确领导下，埋头苦干走实每一步，开拓创新干好每一年，为建设“强富美高”新淮安而努力奋斗!
